# Electrocardiographic findings in peripartum cardiomyopathy

**DOI:** 10.1002/clc.23171

**Published:** 2019-03-29

**Authors:** Michael C. Honigberg, Uri Elkayam, Navin Rajagopalan, Kalgi Modi, Joan E. Briller, Mark H. Drazner, Gretchen L. Wells, Dennis M. McNamara, Michael M. Givertz, for the IPAC Investigators

**Affiliations:** ^1^ Cardiology Division, Massachusetts General Hospital Harvard Medical School Boston Massachusetts; ^2^ Division of Cardiovascular Medicine, Department of Medicine, Keck School of Medicine University of Southern California Los Angeles California; ^3^ Heart Failure and Transplant Cardiology, Newark Beth Israel Medical Center Newark New Jersey; ^4^ Division of Cardiology Louisiana State University Health Sciences Center Shreveport Louisiana; ^5^ Division of Cardiology University of Illinois at Chicago Chicago Illinois; ^6^ Division of Cardiology University of Texas Southwestern Medical Center Dallas Texas; ^7^ Heart and Vascular Institute, University of Pittsburgh Medical Center University of Pittsburgh School of Medicine Pittsburgh Pennsylvania; ^8^ Cardiovascular Division, Brigham and Women's Hospital Harvard Medical School Boston MA

**Keywords:** electrocardiography, maternal‐fetal health, outcomes, peripartum cardiomyopathy

## Abstract

**Background:**

There is limited data on electrocardiographic (ECG) abnormalities and their prognostic significance in women with peripartum cardiomyopathy (PPCM). We sought to characterize ECG findings in PPCM and explore the association of ECG findings with myocardial recovery and clinical outcomes.

**Hypothesis:**

We hypothesized that ECG indicators of myocardial remodeling would portend worse systolic function and outcomes.

**Methods:**

Standard 12‐lead ECGs were obtained at enrollment in the Investigations of Pregnancy‐Associated Cardiomyopathy study and analyzed for 88 women. Left ventricular ejection fraction (LVEF) was measured by echocardiography at baseline, 6 months, and 12 months. Women were followed for clinical events (death, mechanical circulatory support, and/or cardiac transplantation) until 1 year.

**Results:**

Half of women had an “abnormal” ECG, defined as atrial abnormality, ventricular hypertrophy, ST‐segment deviation, and/or bundle branch block. Women with left atrial abnormality (LAA) had lower LVEF at 6 months (44% vs 52%, *P* = 0.02) and 12 months (46% vs 54%, *P* = 0.03). LAA also predicted decreased event‐free survival at 1 year (76% vs 97%, *P* = 0.008). Neither left ventricular hypertrophy by ECG nor T‐wave abnormalities predicted outcomes. A normal ECG was associated with recovery in LVEF to ≥50% (84% vs 49%, *P* = 0.001) and event‐free survival at 1 year (100% vs 85%, *P* = 0.01).

**Conclusions:**

ECG abnormalities are common in women with PPCM, but a normal ECG does not rule out the presence of PPCM. LAA predicted lower likelihood of myocardial recovery and event‐free survival, and a normal ECG predicted favorable event‐free survival.

## 1 INTRODUCTION

Peripartum cardiomyopathy (PPCM) is a dilated cardiomyopathy marked by systolic dysfunction occurring at the end of pregnancy or, more commonly, in the early postpartum period.^1^, ^2^ Globally, incidence appears to be highest in Nigeria (up to 1 in 100 live births)^3^ and Haiti (1 in 300 live births).^4^ In the United States, the incidence of PPCM is rising^5^ and is approximately 4‐fold higher in black women (1 in 1000‐1500 live births) than in Caucasian women (1 in 4000 live births).^2^, ^6^ Other risk factors include older maternal age, multiple gestation, and preeclampsia.^7^, ^8^ The etiology of PPCM remains unclear; proposed mechanisms have included angiogenic factor imbalance, abnormal prolactin cleavage, inflammation, selenium deficiency, and genetic susceptibility.^9^, ^10^, ^11^, ^12^, ^13^, ^14^

More than half of women recover left ventricular (LV) function after PPCM, but a significant proportion is left with chronic heart failure, and some women require ventricular assist device (VAD) implantation or cardiac transplantation. In a recent North American series of 100 women, the Investigations of Pregnancy‐Associated Cardiomyopathy (IPAC) study, 72% of women experienced recovery in LV ejection fraction (LVEF) to >50% by 12 months.^15^ Predictors of persistent LV dysfunction included LVEF <30% or LV end‐diastolic diameter > 6 cm at diagnosis, black race, and late presentation.^15^ Elevation in troponin, B‐type natriuretic peptide, and soluble fms‐like tyrosine kinase‐1 (sFlt‐1) may also portend adverse cardiac remodeling and outcomes.^16^, ^17^, ^18^

There is limited and conflicting data on ECG abnormalities and their significance in women with PPCM. In a Nigerian series of ECGs for 54 cases of PPCM and 77 postpartum women without PPCM, women with PPCM had faster heart rate, longer QRS and QTc intervals, and a higher frequency of ST‐T‐wave abnormalities than controls.^19^ Of note, the authors did not examine the prognostic significance of ECG findings in this cohort. In a series of 78 South African women, 59% had T‐wave abnormalities, 12% had a bundle branch block (BBB), 10% had left atrial abnormality, and 6% had ST‐segment changes on baseline ECG.^20^ Follow‐up was available on only 56% of this cohort, but among women with follow‐up, T‐wave inversions and ST depressions on the presenting ECG were associated with lower LVEF at 6 months.^20^ In a series of 77 women in Beijing with PPCM, neither QRS nor QTc interval nor the frequency of ST depressions differed between women who did and did not recover LV function.^17^ As the phenotypic presentation of PPCM differs across continents,^21^ ECG changes observed in Africa and Asia may not match those seen in North American PPCM patients, for whom there has been no data published on ECG findings to date.

Thus, we sought to characterize ECG findings at PPCM presentation in patients from the United States and Canada in the IPAC cohort and to explore the potential prognostic significance of specific ECG findings in this population. We hypothesized that ECG indicators of myocardial remodeling, such as ventricular hypertrophy and atrial abnormalities, would portend less recovery of systolic function and worse outcomes.

## 2 METHODS

Between 2009 and 2012, 100 women at 30 participating sites in North America with newly diagnosed PPCM were enrolled in the IPAC study up to 13 weeks postpartum. Eligible women were 18 years of age or older, lacked underlying cardiac disease, had an LVEF <45% at enrollment, and had been ruled out for alternate etiologies of cardiomyopathy. All women had an echocardiogram at enrollment, 6, and 12 months, and these studies were reviewed by a core laboratory at the University of Pittsburgh for assessment of ventricular volumes and calculation of ejection fraction. Clinical events, including hospitalizations, mechanical circulatory support, cardiac transplantation, and death, were followed to 12 months postpartum. Institutional review boards at all participating centers approved the protocol and all patients signed informed consent.

Standard left‐sided 12‐lead electrocardiograms (ECGs) were obtained at the time of enrollment and were available for review in 88 of 100 women in the IPAC cohort. For 10 women, a written report of the enrollment ECG was available but not the ECG tracing itself, and two women had tracings of poor quality that were deemed uninterpretable; these 12 subjects were excluded. Features of each ECG tracing (eg, rate, rhythm, intervals, and amplitudes) were systemically analyzed in a blinded fashion by one investigator (M.C.H.), and a second investigator (M.M.G.) validated a random subset of ECG interpretations. The investigators reviewing ECGs were blinded to subject demographics, clinical presentation, echocardiograms, and outcomes.

A normal QRS axis was −30**°** to +90**°**. BBBs were defined per American Heart Association, American College of Cardiology, and Heart Rhythm Society guidelines.^22^ Left atrial abnormality (LAA) was defined as terminal negative deflection of the P‐wave in V_1_ > 40 ms wide and > 1 mm deep. Right atrial abnormality was defined as a P‐wave >2.5 mm tall in II and/or positive initial deflection of the P‐wave in V_1_ > 1.5 mm.^23^ Left ventricular hypertrophy (LVH) was defined using the Sokolow‐Lyon criteria (S in V_1_ plus R in V_5_ or V_6_ ≥ 35 mm and/or R in aVL ≥ 11 mm) or the Cornell voltage criteria for women (S in V_3_ plus R in aVL > 20 mm). Right ventricular hypertrophy (RVH) was defined as R in V_1_ ≥ 7 mm. ST‐segments were coded as depressed if ≥0.5 mm below baseline and as elevated if ≥0.5 mm above baseline. T‐waves were recorded as flattened only if the T‐wave was completely flat. T‐waves were recorded as inverted if they were negatively directed, except for negatively directed T‐waves in III, aVR, or V_1_ with an associated negative QRS complex, which were recorded as normal. The presence of left atrial enlargement and LVH were also assessed by echocardiography. Left atrial enlargement was defined as left atrial diameter ≥40 mm in the parasternal long axis view and LVH as LV posterior wall thickness 12 mm or greater.

Student *t* tests and Fisher exact tests were used to compare continuous and categorical variables between groups, respectively. The Kaplan‐Meier log‐rank analysis was used to estimate event‐free survival, which was defined as survival free from death, mechanical circulatory support, and/or cardiac transplantation. Event‐free survival was compared by characteristics of the ECG at entry, including LAA, LVH, ST segment depression, and a “normal” ECG by the exact log‐rank test. In addition, the LVEF at 6 and 12 months postpartum was compared by ECG characteristics at entry.

## 3 RESULTS

Of the 100 women in the IPAC cohort, 88 had available baseline ECGs. Demographic and clinical characteristics of these women are summarized in Table 1. Mean age was 30 ± 6 years, and 15 (17%) presented with multiple gestation. Diabetes was present in 10 (11%) and hypertension in 38 (43%). At baseline, mean systolic and diastolic blood pressures were 111 ± 17 mm Hg and 70 ± 13 mm Hg, respectively. LVEF at entry was 34% ± 10% and LV end‐diastolic dimension was 5.6 ± 0.7 cm.^15^ By 6 months, LVEF had increased to 51% ± 11%, and by 12 months to 53 ± 11%. Women with an “abnormal ECG” at study enrollment (defined as presence of BBB, ventricular hypertrophy, atrial abnormality, and/or ST‐segment deviation) were more likely to receive inotropes and had a larger LV end‐diastolic dimension (LVEDD) on baseline echocardiogram (58 vs 54 mm, *P* = 0.002). Six women experienced nine major events: four deaths, four LVAD implantations, and one cardiac transplantation. Of women who required an LVAD, two died and one later underwent cardiac transplantation.

**Table 1:** Baseline characteristics of the study population

	All (n = 88)	Normal ECG^a^ (n = 43)	Abnormal ECG^b^ (n = 45)	*P* value
Age (years)	30 ± 6	29 ± 6	31 ± 6	0.21
Gravida	2.9 ± 2.0	2.7 ± 1.9	3.0 ± 2.1	0.55
Parity	2.2 ± 1.3	2.0 ± 1.2	2.4 ± 1.4	0.16
Multiple gestation (%)	17	19	16	0.78
Postpartum (days)	32 ± 25	30 ± 22	33 ± 28	0.52
NHYA class (I/II/III/IV)%	11/49/24/16	16/56/21/7	7/42/27/24	0.008
BMI (kg/m^2^)	29 ± 8	29 ± 6	29 ± 9	0.87
Black (%)	33	30	36	0.65
Diabetes (%)	11	7	16	0.32
Hypertension (%)	43	40	47	0.53
Smoking (%)	33	33	33	0.94
HR exam (beats/min)	86 ± 17	83 ± 16	90 ± 17	0.045
HR ECG (beats/min)	94 ± 24	86 ± 21	102 ± 24	0.001
SBP (mm Hg)	111 ± 17	111 ± 15	110 ± 19	0.82
DBP (mm Hg)	70 ± 13	70 ± 13	70 ± 14	0.99
Beta‐blockers (%)	88	93	82	0.20
ACEIs or ARBs (%)	78	84	73	0.30
Diuretic (%)	68	65	71	0.65
Inotropes (%)	16	5	27	0.007
LVEF at entry (%)	34 ± 10	37 ± 10	32 ± 10	0.02
LVEF at 6 months (%)	51 ± 11	53 ± 8	48 ± 13	0.02
LVEF at 12 months (%)	53 ± 11	55 ± 8	50 ± 13	0.06
LVEDD at entry (cm)	5.6 ± 0.7	5.4 ± 0.5	5.8 ± 0.7	0.002
Recovered^c^ (%)	66	84	49	0.001

Abbreviations: ACEI, angiotensin‐converting enzyme inhibitor; ARB, angiotensin receptor blocker; BMI, body mass index; DBP, diastolic blood pressure; ECG, electrocardiogram HR, heart rate; LVEDD, left ventricular end‐diastolic dimension; LVEF, left ventricular ejection fraction; NYHA, New York Heart Association; SBP, systolic blood pressure.

a “Normal” ECG defined as absence of atrial abnormality, ventricular hypertrophy, ST‐segment deviation, and/or bundle branch block.

b “Abnormal” ECG defined as presence of atrial abnormality, ventricular hypertrophy, ST‐segment deviation, and/or bundle branch block.

c “Recovered” defined as LVEF ≥50% at 12 months.

### 3.1 Electrocardiographic findings

Findings of 12‐lead ECGs at study enrollment are summarized in Table 2. One woman was in atrial fibrillation, and all other subjects were in sinus rhythm; 45 (51%) had a normal sinus rhythm, 37 (42%) showed sinus tachycardia, and 5 (6%) showed sinus bradycardia. Ventricular ectopic beats were observed in 3 subjects. QRS axis was normal in 74 (84%). There was no first‐, second‐, or third‐degree atrio‐ventricular block. Two subjects had a QRS duration >120 ms; one met criteria for left BBB, and the other right BBB. LAA was observed in 15 (17%), right atrial abnormality in 5 (6%), and LVH in 8 (9%) ECGs. ST‐segment depression was seen in 15 (17%) of women, and ST‐segment elevation was seen in 6 (7%). T‐wave abnormalities were common, with flattening seen in 62 (71%) and inversions in 56 (64%). A “normal” ECG, defined as absence of atrial abnormality, ventricular hypertrophy, ST‐segment deviation, or BBB, was present in 43 (49%) of women.

**Table 2:** Features of 12‐lead electrocardiograms at enrollment

Category	ECG feature	Mean ± SD (range) or n (%)
Rate	Ventricular rate	94 ± 24 (52‐146)
Rhythm	Normal sinus rhythm	45 (51.1%)
	Sinus tachycardia	37 (42.0%)
	Sinus bradycardia	5 (5.7%)
	Atrial fibrillation	1 (1.1%)
	Premature atrial contractions	3 (3.4%)
	Premature ventricular contractions	3 (3.4%)
Axis	Normal	74 (84.1%)
	Left	2 (2.3%)
	Right	11 (12.5%)
	Indeterminate	1 (1.1%)
Intervals	PR	143 ± 19 (88‐200)
	QRS	83 ± 14 (60‐146)
	QTc	462 ± 39 (376‐573)
Conduction	Mobitz II	0 (0%)
	Complete heart block	0 (0%)
	Incomplete right bundle branch block	5 (5.7%)
	Complete right bundle branch block	1 (1.1%)
	Incomplete left bundle branch block	1 (1.1%)
	Complete left bundle branch block	1 (1.1%)
	Left anterior fascicular block	1 (1.1%)
	Left posterior fascicular block	0 (0%)
Chambers	Right atrial abnormality	5 (5.7%)
	Left atrial abnormality	15 (17.0%)
	Right ventricular hypertrophy	0 (0%)
	Left ventricular hypertrophy	8 (9.1%)
Repolarization	ST‐segment depression	15 (17.0%)
	ST‐segment elevation	6 (6.8%)
	T‐wave flattening	62 (70.5%)
	T‐wave inversion	56 (63.6%)

### 3.2 Prediction of left ventricular recovery and event‐free survival

Tables 3 and 4 show the trajectory of LV function for women with and without various ECG and echocardiographic findings. As reported previously,^15^ heart rate at enrollment was not correlated with LV recovery (*P* = 0.40 at 6 months and *P* = 0.26 at 12 months).

**Table 3:** Left ventricular ejection fraction trajectory for women with and without left atrial abnormality and left ventricular hypertrophy by electrocardiogram and echocardiogram

	Left ventricular ejection fraction (%)							
	Abnormality detected by ECG				Abnormality detected by echocardiogram^b^			
		Enrollment	6 months	12 months		Enrollment	6 months	12 months
Left atrial abnormality^a^	Yes (n = 15)	31 ± 12	44 ± 14	46 ± 16	Yes (n = 35)	32 ± 11	46 ± 14	47 ± 14
	No (n = 72)	35 ± 9	52 ± 9	54 ± 9	No (n = 52)	36 ± 9	54 ± 7	56 ± 7
	*P* value	0.185	0.020	0.026	*P* value	0.045	0.003	0.001
Left ventricular hypertrophy	Yes (n = 8)	27 ± 6	45 ± 10	52 ± 11	Yes (n = 7)	32 ± 11	45 ± 20	47 ± 17
	No (n = 80)	35 ± 10	51 ± 11	53 ± 11	No (n = 79)	35 ± 10	52 ± 9	54 ± 10
	*P* value	0.029	0.105	0.736	*P* value	0.553	0.145	0.142

a Excludes one subject in atrial fibrillation on the enrollment ECG.

b Left atrial abnormality by echocardiogram defined as left atrial diameter greater than 40 mm. Left ventricular hypertrophy by echocardiogram defined as posterior wall thickness 12 mm or greater.

**Table 4:** Left ventricular ejection fraction trajectory for women with and without electrocardiographic findings

ECG findings		Left ventricular ejection fraction (%)		
		Enrollment	6 months	12 months
Normal^a^	Yes (n = 43)	37 ± 10	53 ± 8	55 ± 8
	No (n = 45)	32 ± 10	48 ± 13	50 ± 13
	*P* value	0.020	0.021	0.61
ST‐segment depression	Yes (n = 15)	30 ± 8	40 ± 16	45 ± 18
	No (n = 73)	35 ± 10	52 ± 9	54 ± 9
	*P* value	0.102	0.003	0.025
T‐wave inversions	Yes (n = 56)	33 ± 10	51 ± 11	53 ± 11
	No (n = 32)	37 ± 9	50 ± 10	54 ± 9
	*P* value	0.074	0.708	0.756
T‐wave flattening	Yes (n = 62)	34 ± 10	49 ± 12	51 ± 12
	No (n = 26)	35 ± 10	54 ± 7	57 ± 5
	*P* value	0.702	0.103	0.030

a “Normal” ECG defined as absence of bundle branch block, ventricular hypertrophy, atrial abnormality, or ST‐segment deviation.

**Figure 1:**
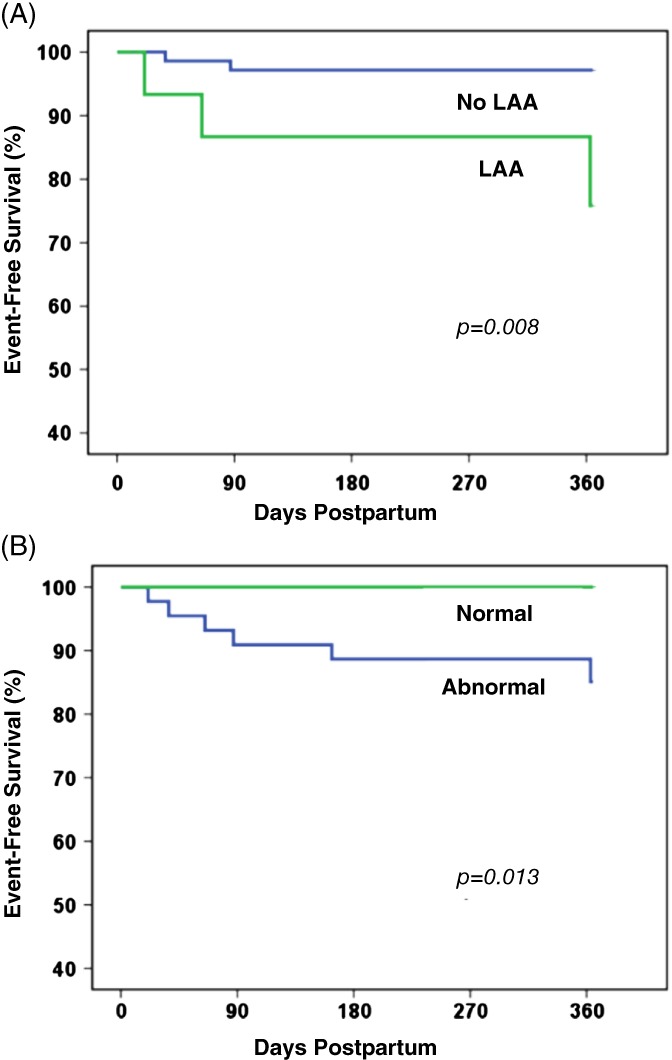
(A) Survival free from mechanical circulatory support, cardiac transplantation, and/or death for women with and without left atrial abnormality (LAA) on electrocardiogram (ECG) at study enrollment. (B) Survival free from mechanical circulatory support, cardiac transplantation, and/or death for women with a “normal” ECG (no atrial abnormality, ventricular hypertrophy, ST‐segment deviation, or bundle branch block) or “abnormal” ECG at study enrollment

LAA by ECG was specific (96%) but not sensitive (38%) for left atrial enlargement by echocardiogram. The presence of LAA on ECG was associated with lower LVEF at 6 months (44% vs 52%, *P* = 0.02) and 12 months (46% vs 54%, *P* = 0.03); these findings are almost identical to those comparing women with and without left atrial enlargement by echocardiogram (LVEF 46% vs 54% at 6 months (*P* = 0.003), and 47% vs 56% at 12 months (*P* = 0.001). As shown in Figure 1A, the presence of LAA by ECG additionally predicted decreased event‐free survival at 1 year (76% vs 97%, *P* = 0.008).

LVH was evident by ECG criteria in eight women (9%) and was associated with a lower LVEF at entry (27% vs 35%, *P* = 0.03), but not at 6 months (45% vs 51%, *P* = 0.10) or 12 months (52% vs 53%, *P* = 0.74). Of note, there was no concordance between identification of LVH by ECG and echocardiography (defined as LV posterior wall thickness 12 mm or greater), with only one woman meeting criteria by both modalities. LVH by echocardiogram was seen in seven women and was not predicative of LVEF at entry (*P* = 0.55), 6 months (*P* = 0.14), or 12 months (*P* = 0.14). While there was a trend toward less recovery of EF in women with LVH, the small number of these women provides limited power to detect differences. LVH was not associated with event‐free survival at 1 year when assessed by either modality (*P* = 0.51 for LVH by ECG, *P* = 0.47 for LVH by echocardiogram).

The presence of ST‐segment depressions correlated with decreased LV recovery at 6 months (LVEF 40% vs 52%, *P* = 0.003) and 12 months (45% vs 54%, *P* = 0.025), but was not predictive of event‐free survival (*P* = 0.92). T‐wave abnormalities were not predictive of myocardial recovery or event‐free survival, except for a small difference in LVEF at 12 months in women with and without T‐wave flattening (51% vs 57%, *P* = 0.03).

The presence of a “normal” ECG predicted a significantly higher LVEF at 6 months (53% vs 48%, *P* = 0.02), which was no longer significant at 12 months (55% vs 50%, *P* = 0.06). However, a normal ECG was associated with recovery to an LVEF ≥50% by 12 months (84% vs 49%, *P* = 0.001). Event‐free survival at 1 year was significantly better (100% vs 85%, *P* = 0.013, Figure 1B), as no events occurred in women with a normal ECG at entry.

## 4 DISCUSSION

We present here electrocardiographic findings in a large, prospective North American cohort of women with PPCM. To the best of our knowledge, this is the largest collection of ECGs in PPCM reported to date, the first report in North America, and the first to explore the association between ECG findings and clinical outcomes (ie, death, mechanical circulatory support, and transplantation). Among prior studies of the ECG in PPCM, Karaye et al^19^ did not study prognosis in relation to ECG findings, Tibazarwa et al.^20^explored fewer ECG parameters in relation to outcomes with incomplete follow‐up, and Li et al^17^ examined only QRS and QTc.

In our cohort, the majority of ECGs at study enrollment revealed sinus rhythm, a normal QRS axis, and normal intervals. LAA was seen in 17% of women and predicted both decreased recovery of LV function and decreased event‐free survival. This finding is similar to that reported in a study of 468 patients with heart failure after non‐ST‐elevation myocardial infarction, in which electrocardiographic LAA was associated with higher risk of recurrent heart failure and mortality.^24^ As noted, LAA by ECG was only 38% sensitive for left atrial enlargement as assessed by echocardiography. Prior work has suggested modest sensitivity of electrocardiographic LAA for left atrial enlargement measured by echocardiogram, with estimates ranging from 30%‐75% when using terminal negative P‐wave duration in V_1_ > 40 ms.^25^, ^26^ It is unclear from the available data whether electrical LAA is associated with adverse events because it indicates more severe left atrial enlargement or whether it is associated with adverse clinical events independent of left atrial size.

Importantly, a “normal” ECG was seen in 49% of women in our series and portended a higher likelihood of recovering LV systolic function and survival free from mechanical circulatory support or transplant at 1 year. Of note, Tibazarwa et al^20^ reported a normal ECG only in 4% of women in their series. Some, but not all, of this discrepancy arises from the fact that we did not include T‐wave abnormalities in our definition of “abnormal.” We made this decision because T‐wave abnormalities were present in the majority of our cohort, thus, their inclusion would have diminished the predictive capability of an abnormal ECG, and because such T‐wave changes may also be seen in normal pregnancy. Indeed, T‐wave abnormalities were the most common noted abnormality but, in contrast to findings reported by Tibazarwa et al, were not predictive of adverse clinical outcomes in our cohort. The association of an abnormal ECG with a larger LVEDD and higher likelihood of requiring inotropic support align with our hypothesis that ECG abnormalities may reflect pathologic remodeling that has already occurred at the time of diagnosis and may therefore indicate more severe cardiomyopathy. The relatively high proportion of women in our series with a normal ECG or only non‐specific T‐wave abnormalities demonstrates that ECG has poor sensitivity as a screening tool for the detection of PPCM in the North American setting.

One limitation of the current study is the absence of follow‐up ECGs to determine the natural history of abnormalities detected on baseline 12‐lead ECG. In addition, although a small portion of women with PPCM are diagnosed in the final weeks of pregnancy,^27^ all women in the IPAC cohort were enrolled postpartum. Pregnancy itself is associated with ECG changes, chiefly increased heart rate, change in QRS axis (most commonly a leftward axis), non‐specific ST‐segment changes, and change in T‐wave axis,^28^, ^29^ although normal pregnancy is not known to be associated with changes in conduction or chamber enlargement. Our analysis thus likely generalizes to women diagnosed with PPCM during pregnancy, but future studies should validate our findings in women who are still pregnant at the time of diagnosis.

## 5 CONCLUSIONS

ECG changes are common at the time of presentation with PPCM and can predict likelihood of LV recovery. A normal ECG, however, does not rule out the presence of PPCM. LAA predicted lower likelihood of LV recovery and event‐free‐survival, and a normal ECG predicted favorable outcomes. Future research should integrate ECG findings with clinical, imaging, and biomarker data to help determine prognosis and guide management of this condition.

## References

[1] Asad ZUA , Maiwand M , Farah F , Dasari TW . Peripartum cardiomyopathy: a systematic review of the literature. Clin Cardiol. 2018;41:693‐697. 29749620 10.1002/clc.22932 PMC6489815

[2] Honigberg MC , Givertz MM . Peripartum cardiomyopathy. BMJ. 2019;364:k5287. 30700415 10.1136/bmj.k5287

[3] Isezuo SA , Abubakar SA . Epidemiologic profile of peripartum cardiomyopathy in a tertiary care hospital. Ethn. Dis. 2007;17:228‐223. 17682350

[4] Fett JD , Christie LG , Carrway RD , et al. Five‐year prospective study of the incidence and prognosis of peripartum cardiomyopathy at a single institution. Mayo Clin Proc. 2005;80:1602‐1606. 16342653 10.4065/80.12.1602

[5] Kolte D , Khera S , Aronow WS , et al. Temporal trends in the incidence and outcomes of peripartum cardiomyopathy in the United States: a nationwide population‐based study. J Am Heart Assoc. 2014;3:e001056. 24901108 10.1161/JAHA.114.001056 PMC4309108

[6] Brar SS , Khan SS , Sandhu GK , et al. Incidence, mortality, and racial differences in peripartum cardiomyopathy. Am J Cardiol. 2007;100:302‐304. 17631087 10.1016/j.amjcard.2007.02.092

[7] Arany Z , Elkayam U . Peripartum cardiomyopathy. Circulation. 2016;133:1397‐1409. 27045128 10.1161/CIRCULATIONAHA.115.020491

[8] Lee S , Cho GJ , Park GU , et al. Incidence, risk factors, and clinical characteristics of peripartum cardiomyopathy in South Korea. Circ Heart Fail. 2018;11:e004134. 29626099 10.1161/CIRCHEARTFAILURE.117.004134

[9] Patten IS , Rana S , Shahul S , et al. Cardiac angiogenic imbalance leads to peripartum cardiomyopathy. Nature. 2012;485:333‐338. 22596155 10.1038/nature11040 PMC3356917

[10] Hilfiker‐Kleiner D , Kaminski K , Podewski E , et al. A cathepsin D‐cleaved 16 kDa form of prolactin mediates postpartum cardiomyopathy. Cell. 2007;128:589‐600. 10.1016/j.cell.2006.12.036 17289576

[11] Sliwa K , Fett J , Elkayam U . Peripartum cardiomyopathy. Lancet. 2006;368:687‐693. 16920474 10.1016/S0140-6736(06)69253-2

[12] Karaye KM , Yahaya IA , Lindmark K , Henein M . Serum selenium and ceruloplasmin in Nigerians with peripartum cardiomyopathy. Int J Mol Sci. 2015;16:7644‐7654. 25853263 10.3390/ijms16047644 PMC4425040

[13] Ware JS , Li J , Mazaika E , et al. Shared genetic predisposition in peripartum and dilated cardiomyopathies. N Engl J Med. 2016;374:233‐241. 26735901 10.1056/NEJMoa1505517 PMC4797319

[14] Morales A , Painter T , Li R , et al. Rare variant mutations in pregnancy‐associated or peripartum cardiomyopathy. Circulation. 2010;121:2176‐2182. 20458009 10.1161/CIRCULATIONAHA.109.931220 PMC2900861

[15] McNamara DM , Elkayam U , Alharethi R , et al. Clinical outcomes for peripartum cardiomyopathy in North America: results of the IPAC study (Investigations of Pregnancy‐Associated Cardiomyopathy). J Am Coll Cardiol. 2015;66:905‐914. 26293760 10.1016/j.jacc.2015.06.1309 PMC5645077

[16] Hu CL , Li YB , Zou YG , et al. Troponin T measurement can predict persistent left ventricular dysfunction in peripartum cardiomyopathy. Heart. 2007;93:488‐490. 17065185 10.1136/hrt.2006.087387 PMC1861492

[17] Li W , Li H , Long Y . Clinical characteristics and long‐term predictors of persistent left ventricular systolic dysfunction in peripartum cardiomyopathy. Can J Cardiol. 2016;32:362‐368. 26586094 10.1016/j.cjca.2015.07.733

[18] Damp J , Givertz MM , Semigran M , et al. Relaxin‐2 and soluble Flt1 levels in peripartum cardiomyopathy: results of the multicenter IPAC Study. JACC Heart Fail. 2016;4:380‐388. 26970832 10.1016/j.jchf.2016.01.004 PMC4851559

[19] Karaye KM , Lindmark K , Henein MY . Electrocardiographic predictors of peripartum cardiomyopathy. Cardiovasc J Afr. 2016;27:66‐70. 27213852 10.5830/CVJA-2015-092 PMC4928165

[20] Tibazarwa K , Lee G , Mayosi B , Carrington M , Stewart S , Sliwa K . The 12‐lead ECG in peripartum cardiomyopathy. Cardiovasc J Afr. 2012;23:322‐329. 22337203 10.5830/CVJA-2012-006 PMC3734749

[21] Sliwa K , Mebazaa A , Hilfiker‐Kleiner D , et al. Clinical characteristics of patients from the worldwide registry on peripartum cardiomyopathy (PPCM): EURObservational Research Programme in conjunction with the Heart Failure Association of the European Society of Cardiology Study Group on PPCM. Eur J Heart Fail. 2017;19:1131‐1141. 28271625 10.1002/ejhf.780

[22] Surawicz B , Childers R , Deal BJ , et al. AHA/ACCF/HRS recommendations for the standardization and interpretation of the electrocardiogram: part III: intraventricular conduction disturbances: a scientific statement from the American Heart Association Electrocardiography and Arrhythmias Committee, Council on Clinical Cardiology; the American College of Cardiology Foundation; and the Heart Rhythm Society: endorsed by the International Society for Computerized Electrocardiology. Circulation. 2009;119:e235‐e240. 19228822 10.1161/CIRCULATIONAHA.108.191095

[23] Hancock EW , Deal BJ , Mirvis DM , et al. AHA/ACCF/HRS recommendations for the standardization and interpretation of the electrocardiogram: part V: electrocardiogram changes associated with cardiac chamber hypertrophy: a scientific statement from the American Heart Association Electrocardiography and Arrhythmias Committee, Council on Clinical Cardiology; the American College of Cardiology Foundation; and the Heart Rhythm Society: endorsed by the International Society for Computerized Electrocardiology. Circulation. 2009;119:e251‐e261. 19228820 10.1161/CIRCULATIONAHA.108.191097

[24] Ariyarajah V , Malinski M , Zieroth S , Harizi R , Morris A , Spodick DH . Risk stratification for recurrent heart failure in patients post‐myocardial infarction with electrocardiographic and echocardiographic left atrial abnormality. Am J Cardiol. 2008;101:1373‐1378. 18471444 10.1016/j.amjcard.2008.01.013

[25] Hazen MS , Marwick TH , Underwood DA . Diagnostic accuracy of the resting electrocardiogram in detection and estimation of left atrial enlargement: an echocardiographic correlation in 551 patients. Am Heart J. 1991;122:823‐828. 1831587 10.1016/0002-8703(91)90531-l

[26] Lee KS , Appleton CP , Lester SJ , et al. Relation of electrocardiographic criteria for left atrial enlargement to two‐dimension echocardiographic left atrial volume measurements. Am J Cardiol. 2007;99:113‐118. 17196473 10.1016/j.amjcard.2006.07.073

[27] Elkayam U . Clinical characteristics of peripartum cardiomyopathy in the United States: diagnosis, prognosis, and management. J Am Coll Cardiol. 2011;58:659‐670. 21816300 10.1016/j.jacc.2011.03.047

[28] Carruth JE , Mivis SB , Brogan DR , et al. The electrocardiogram in normal pregnancy. Am Heart J. 1981;102:1075‐1078. 7315707 10.1016/0002-8703(81)90497-x

[29] Oram S , Holt M . Innocent depression of the S‐T segment and flattening of the T‐wave during pregnancy. J Obstet Gynaecol Br Emp. 1961;68:765‐770. 14482159 10.1111/j.1471-0528.1961.tb02807.x

